# Effect of Catechin on Hepatotoxicity Induced by Combined Doxorubicin and Paclitaxel Treatment

**DOI:** 10.1155/jt/6775839

**Published:** 2025-09-03

**Authors:** Shayan Shwan Mohammed-Rashid, Hiewa Othman Dyary

**Affiliations:** Department of Basic Sciences, College of Veterinary Medicine, University of Sulaimani, New Sulaimani, Street 27, Sulaymaniyah 46001, Kurdistan Region, Iraq

**Keywords:** antioxidant, catechin, chemotherapy, doxorubicin, hepatotoxicity, oxidative stress, paclitaxel

## Abstract

Chemotherapy-induced hepatotoxicity remains a significant challenge in cancer treatment, limiting the clinical use of potent anticancer agents like doxorubicin (DOX) and paclitaxel (PAC). This study investigated the hepatoprotective effects of catechin (CAT), a natural flavonoid antioxidant, against DOX- and PAC-induced liver toxicity. Male Wistar rats were divided into five groups: control, DOX + PAC-treated, CAT-only, and two groups receiving CAT (20 or 40 mg/kg) in combination with DOX + PAC. Hepatic function was assessed through liver enzyme levels, oxidative stress biomarkers, and histopathological examination. Results showed that DOX + PAC treatment significantly elevated serum levels of ALT, AST, and ALP, indicating hepatocellular damage. Oxidative stress markers, including malondialdehyde (MDA) and nuclear factor kappa B (NF-κB), were also increased, while antioxidant defenses such as glutathione (GSH) and catalase were depleted. CAT coadministration, particularly at 40 mg/kg, markedly reduced oxidative damage, restored hepatic enzyme levels, and mitigated histopathological alterations, including congestion, hepatocyte degeneration, and inflammatory infiltration. Moreover, CAT reduced NF-κB expression, suggesting an anti-inflammatory effect. These findings demonstrate that CAT effectively protects against DOX- and PAC-induced hepatotoxicity by enhancing antioxidant defense mechanisms and reducing inflammation. Given its hepatoprotective potential, CAT may serve as a complementary therapeutic strategy to enhance chemotherapy tolerance.

## 1. Introduction

Side effects, such as nephrotoxicity, neurotoxicity, and cardiotoxicity, often limit the efficacy of chemotherapeutics. Besides these toxicities, hepatotoxicity is a critical limiting factor when using anticancer agents [1]. Hence, oncologists and hepatologists must collaborate to monitor patients taking cancer therapies and intervene to prevent permanent liver damage [2].

Since chemotherapy is nearly always accompanied by cytotoxicity, combination therapy is a logical approach to improve responsiveness and tolerability while reducing resistance [3]. The most widely used chemotherapeutic medications in clinical use are taxanes and anthracyclines due to their superior antitumor efficacy against various solid malignancies [4, 5]. The most commonly utilized anticancer agents are paclitaxel (PAC) and doxorubicin (DOX). The potential to combine the two medications is made possible by their distinct mechanisms of action [6].

PAC is a potent anticancer drug from the taxane family, widely used to treat ovarian, breast, lung, and other solid tumors [7, 8]. PAC demonstrates strong antitumor activity by stabilizing microtubules, preventing their depolymerization, and promoting their assembly from tubulin dimers [4]. This mechanism disrupts normal cell division, slowing cell cycle progression, mitosis, and the proliferation of cancer cells. PAC facilitates tubulin polymerization rather than inhibiting it [9].

DOX is a member of the anthracycline class and is an effective treatment for many solid tumors [10]. It intercalates between DNA base pairs, disrupting DNA strands and inhibiting the formation of both DNA and RNA. DOX inhibits topoisomerase II, damages DNA, and induces apoptosis [11]. DOX is used to treat several cancers, like sarcomas, carcinomas, breast cancer, and hematological malignancies [12].

Combinations of taxanes and DOX are frequently used to treat solid tumors, including metastatic and advanced breast cancer. These medications are highly effective and do not exhibit evidence of cross-resistance [13]. However, even though the use of PAC and DOX in combination for cancer therapy is progressing, many adverse effects still arise, including hepatotoxicity [13, 14].

PAC produces reactive oxygen species (ROS), which cause mitochondrial dysfunction, resulting in the release of cytochrome C into the cytoplasm and stimulating apoptosis [15]. PAC triggers oxidative stress and decreases antioxidants. It also increases liver enzymes and causes renal dysfunction due to the oxidative stress it induces [16]. PAC exacerbates liver damage and induces hepatocellular necrosis, which can be fatal [17]. It also induces inflammatory reactions, significantly increasing proinflammatory cytokines [18].

The primary mechanism of anthracycline toxicity in both cancerous and normal cells is its interaction with DNA, as exemplified by DOX [19]. The DOX-induced hepatotoxicity is mediated by oxidative stress through the liberation of ROS during DOX metabolism in the liver, leading to mitochondrial dysfunction and inflammation [20]. The outcome is hepatocellular destruction, with the release of enzymes into the circulation [21]. DOX disrupts the balance between ROS and antioxidants, causing lipid peroxidation and protein oxidation in tissue and resulting in tissue injury. Excess free radicals lead to oxidative stress, the primary cause of several degenerative diseases [22].

In general, an antioxidant—natural or synthetic—reduces the harmful effects of ROS and reactive nitrogen oxide species on biomolecules, primarily proteins, lipids, and DNA [23]. Endogenous antioxidants like catalase and superoxide dismutase (SOD) reduce the effects of ROS. However, large quantities of ROS can overwhelm these antioxidants. Numerous investigations have indicated that liver damage caused by hazardous drugs like DOX is often linked to oxidative stress, lipid peroxidation, free radicals, and inflammatory processes. It has been suggested that therapeutically regulating these mediators is necessary to avoid DOX-induced toxicities in different organs [24].

Catechin (CAT), a member of the flavonoid family of polyphenolic compounds, has shown considerable promise in combating various diseases and enhancing general health. Medical examinations have demonstrated their effectiveness in treating various illnesses, such as upper respiratory infections, neuroprotection, and cardioprotective effects. It may also treat various forms of liver disease, though most studies link its potential to antioxidant and radical scavenging effects. Studies conducted both in vivo and in vitro have shown that CAT protects the integrity and function of the liver [25, 26]. Considering the antioxidant properties of CAT, we aimed to alleviate DOX- and PAC-induced oxidative stress by CAT in a controlled experimental study.

## 2. Materials and Methods

### 2.1. Reagents and Kits Used in the Study

The drugs and chemicals used in the study included the following: DOX (DOXO-cell), produced by Stadpharm (Germany), PAC, manufactured by Sobhan Oncology (Iran), xylazine (Beltazyn 2%) from Beltavetpharm (Türkiye), ketamine (Keta-Control) from Doga Ilac (Türkiye), and formalin, from CDH (China).

The following kits were employed (manufactured by Bioassay Technology Laboratory, China): rat nuclear factor kappa B (NF-κB) ELISA kit (Cat. No. E0287Ra), rat catalase ELISA kit (Cat. No. E0869Ra), rat total antioxidant capacity (TAOC) ELISA kit (Cat. No. E3901Ra), rat glutathione (GSH) ELISA kit (Cat. No. EA0113Ra), and rat malondialdehyde (MDA) ELISA kit (Cat. No. E0156Ra).

### 2.2. Animals and Housing

Albino Wistar rats with an average weight of 153.33 g at arrival were used in the study. They were housed at the Research Center's Animal House, located within the College of Veterinary Medicine at the University of Sulaimani. The rats were housed in 40 × 30 × 20 cm^3^ polypropylene cages and provided with water and feed. Wood shavings were used as the cages' bedding and were changed when required. The room temperature was around 25°C, ventilation was controlled, and a 12 h light/dark cycle was followed. The rats were acclimated for 2 weeks before the experiment began.

### 2.3. Treatment Procedures

Thirty rats (8–12-week-old male albino Wistar) were divided randomly into five equal groups, and each rat was numbered on its tail with a permanent marker. Group 1 served as the negative control (NC) and was left untreated throughout the study. Group 2 (DOX + PAC) was a positive control (PC), and the rats were injected intraperitoneally on the ninth day with a dose of DOX (manufactured by Stadpharm, Germany) and PAC (manufactured by Sobhan Oncology, Rasht, Iran) (10.0 mg/kg each) [16]. The rats in Group 3 (CAT) were administered 40 mg/kg of CAT (manufactured by Macklin, China) by gavage for 10 consecutive days [27, 28]. CAT was dissolved in water before administration. Group 4 (DOX + PAC + CAT (HD)) rats were administered 40 mg/kg CAT orally for 10 days and injected with DOX + PAC (10 mg/kg each) on the ninth day [16, 27, 28]. Group 5 (DOX + PAC + CAT (LD)) rats were administered 20 mg/kg CAT orally for 10 days and were injected with DOX + PAC (10 mg/kg each) on the ninth day [16, 29]. The drugs were given according to each rat's weight.

### 2.4. Animal Euthanasia and Collection of Blood and Liver Samples

On day 11 of the experiment, the rats were weighed and then anesthetized with an intramuscular mixture of ketamine (100 mg/kg manufactured by Doga Ilac, Türkiye) and xylazine (20 mg/kg manufactured by Beltavetpharm, Türkiye). After opening the chest cavity, blood was taken from the heart and put into plain test tubes and tubes with anticoagulant. Blood in plain tubes was used to collect serum samples for serological analysis. Moreover, the blood collected in tubes with an anticoagulant was used to measure the hematological parameters.

The livers were soaked with normal saline, dried with sterile gauze, weighed using a digital balance, and excised. Two grams of liver tissue was homogenized in 18 mL of phosphate-buffered saline (PBS) and stored at −80°C for oxidative stress analysis by measuring GSH, catalase, MDA, NF-κB, and TAOC levels. At the same time, the remaining portion of the liver was put in 10% formalin for histopathological examination.

### 2.5. Determination of Liver Function Biomarkers in Serum

The serum samples were centrifuged using a microcentrifuge (Hettich Zentrifugen-EBA20, Germany). Then, the serum levels of alkaline phosphatase (ALP), aspartate transaminase (AST), alanine transaminase (ALT), total bilirubin (TB), gamma-glutamyl transferase (GGT), and albumin were measured with Cobas c 311 (Roche/Hitachi, Switzerland).

### 2.6. Liver Oxidative Stress and Antioxidant Biomarkers' Analysis

An MDA ELISA kit (Bioassay Technology Laboratory/China) was used to test liver tissue for MDA, an indicator of oxidative stress and cell membrane damage. A rat TAC ELISA kit (Bioassay Technology Laboratory/China) was used to test for TAOC in liver tissue. This test helps evaluate the liver's ability to counteract oxidative stress by neutralizing free radicals. Moreover, a rat NF-κB ELISA kit (Bioassay Technology Laboratory/China) test was used to assess the NF-κB activation level, which is critical in inflammation, immune response, oxidative stress, and disease progression.

Furthermore, a rat GSH-ELISA kit (Bioassay Technology Laboratory/China) was used to measure GSH, which is critical in the antioxidant defense system and detoxification. GSH helps neutralize ROS, detoxify harmful compounds, and protect liver cells from oxidative damage. Also, a rat catalase ELISA kit (Bioassay Technology Laboratory/China) was used to measure catalase enzyme levels, which play a crucial role in the antioxidant defense system by breaking down hydrogen peroxide (H_2_O_2_) into water and oxygen. This helps protect liver cells from oxidative stress and damage.

The procedures were conducted following the manufacturer's instructions.

### 2.7. Hepatic Tissue Histopathological Examination

A previous study by Hassan et al. [30] was followed to conduct the histopathological examination. Five-micrometer-thick sections were stained with hematoxylin and eosin (H&E), coverslipped, and then read under a standard light microscope (Leica, Japan).

Several parameters were studied using a light microscope (Motic, Japan) to grade and score pathologic alterations in the hepatic tissues (Table 1). The parameters included changes in architecture, portal triads, hepatocytes, sinusoids, inflammation, degeneration, necrosis, and fatty changes in the hepatic tissue.

The alterations were classified into four scores as the following: grade 0 (normal histological findings (0%)), grade 1 (mild change in up to 25%; initiation of change), grade 2 (moderate change in 26%–50%), grade 3 (moderate-severe change in 51%–75%), and grade 4 (severe change in > 75%). The histological alterations were examined by blind analysis.

### 2.8. Ethical Statement

The scientific and ethical committees at the College of Veterinary Medicine, University of Sulaimani, approved the study protocol as indicated by the approval number AUP-2024-18.

### 2.9. Statistical Analysis

The result data were shown as the mean ± standard error (SEM). A one-way analysis of variance was used to determine the presence of differences in the overall data, and Duncan's multiple range test (post hoc analysis) was used to identify significant differences between the groups. A *p* value of 0.05 was used to determine significant differences.

## 3. Results

### 3.1. Animal and Liver Weights

**Figure 1:**
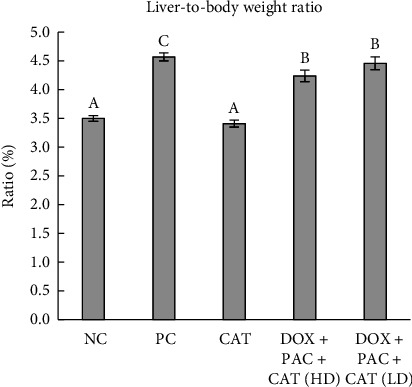
Liver-to-body weight ratios on day 11. Values represent the means of six rats per group (columns) ± SEM (error bars). Different letters (A, B, C) denote significant differences between the groups at *p* < 0.05.

The rats' weights ranged between 236.5 g and 248.3 g 9 days after the experiment (Figure 1). The weights showed no significant differences among groups on day 9 and day 11 of the study (*p* > 0.05). The liver weights in the PC group were significantly higher than those of the other groups on day 11 (Figure 1), indicating acute hepatotoxicity due to DOX and PAC administration, compared to the groups not receiving the anticancer drugs. The liver-to-body weight ratio followed a similar trend, with the PC group displaying a significant increase compared to the NC and CAT-treated groups. Notably, administration of 40 mg/kg CAT with DOX and PAC significantly mitigated this effect, suggesting its hepatoprotective potential.

### 3.2. Hematological Parameters

The total and differential leukocyte counts on day 11 of the study are shown in Table 2. Total leukocyte counts were significantly lower in the PC group than in the NC and CAT-treated groups on day 11. Despite CAT administration, leukocytopenia persisted in the groups that received DOX + PAC combined with either 40 or 20 mg/kg CAT.

No significant differences were observed in erythrocyte counts, hemoglobin, hematocrit, MCV, MCH, and MCHC (Table 3). However, thrombocyte counts were significantly lower in the PC group and in both CAT + DOX + PAC groups compared to the NC and CAT-only groups, indicating a potential suppressive effect of the anticancer drugs on platelet production.

### 3.3. Liver Function Biomarkers in Serum

**Figure 2:**
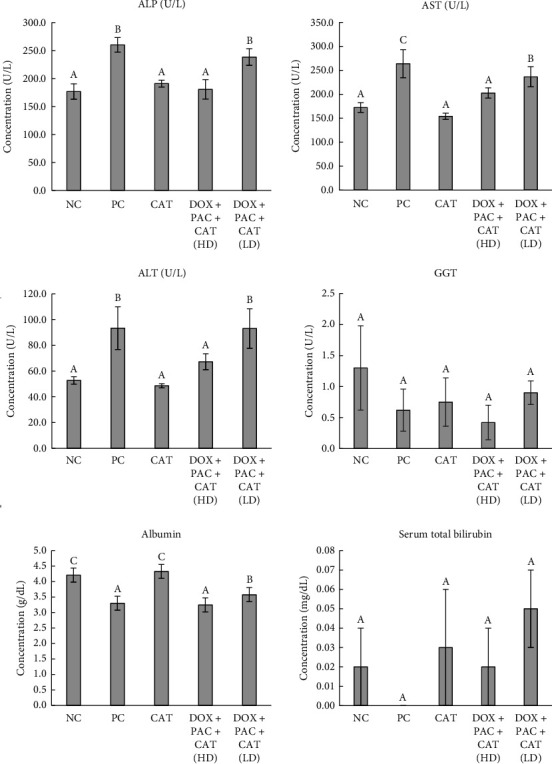
Serum levels of ALP, AST, ALT, GGT, albumin, and total bilirubin. Values represent the means of six rats per group (columns) ± SEM (error bars). Different letters (A, B, C) denote significant differences between the groups at *p* < 0.05.

The serum levels of ALP, AST, ALT, GGT, albumin, and TB are illustrated in Figure 2. Serum levels of ALP, AST, and ALT were significantly elevated in the PC group compared to the NC group, highlighting hepatocellular damage. No differences were observed between the NC and CAT groups. Notably, administration of 40 mg/kg CAT significantly reduced these enzyme levels, suggesting a protective effect against drug-induced liver injury. No significant changes were observed in GGT or TB levels among the groups. However, serum albumin concentrations were significantly lower in the PC group compared to the NC and CAT-only groups, further indicating liver dysfunction. The reduction in albumin levels persisted in the CAT + DOX + PAC groups, albeit to a lesser extent, particularly with the higher dose of CAT.

### 3.4. Liver Oxidative Stress and Antioxidant Biomarkers

**Figure 3:**
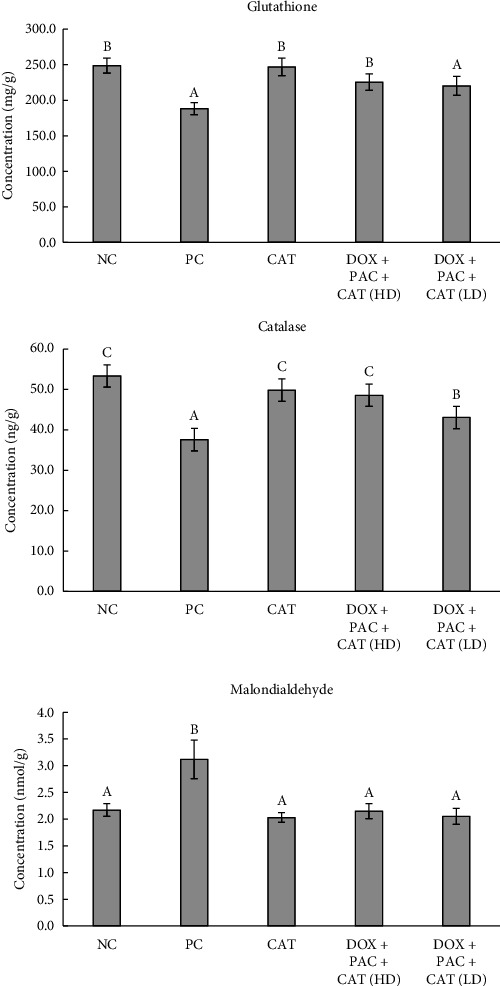
Levels of glutathione, catalase, and malondialdehyde in the hepatic tissue. Values represent the means of six rats per group (columns) ± SEM (error bars). Different letters (A, B) denote significant differences between the groups at *p* < 0.05.

The GSH, catalase, and MDA levels are shown in Figure 3. GSH and catalase levels were significantly lower in the PC group than in the NC group, while MDA levels were elevated, indicating oxidative stress. Administration of 40 mg/kg CAT significantly increased GSH and catalase levels while reducing MDA levels, suggesting an attenuation of oxidative damage. NF-κB expression was significantly higher in the PC group, indicative of inflammation, but was reduced with 40 mg/kg CAT treatment.

**Figure 4:**
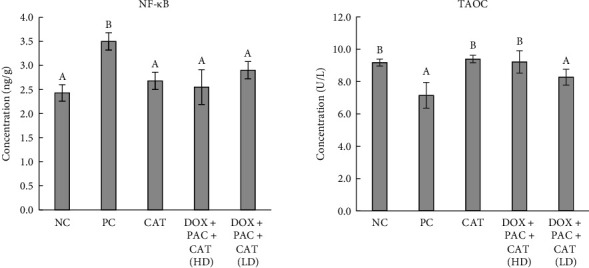
Concentrations of NF-κB and TAOC in the hepatic tissue. Values represent the means of six rats per group (columns) ± SEM (error bars). Different letters (A, B) denote significant differences between the groups at *p* < 0.05.

The concentrations of NF-κB and TAOC are shown in Figure 4. NF-κB levels were higher in the PC than in the NC, CAT, and the group that received 40 mg/kg CAT + DOX + PAC, indicating that CAT administration at a higher dose prevented a rise in NF-κB. TAOC was significantly lower in the PC group but improved with high-dose CAT treatment.

### 3.5. Histopathologic Examination

**Figure 5:**
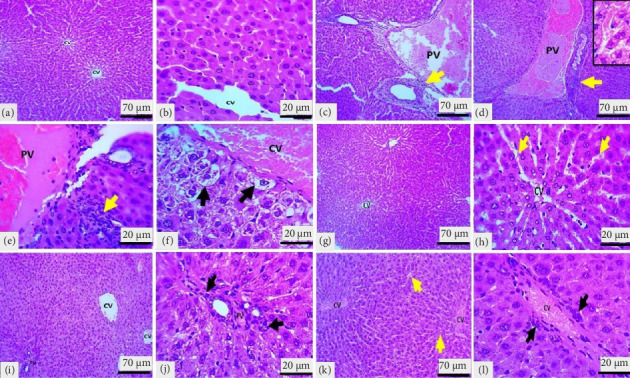
Light microscopic sections of the liver in the different experimental groups. The liver in the NC group (a, b) shows the typical, organized liver arrangement with intact hepatocytes and sinusoidal capillaries. Light microscopic sections of the liver in the PC group (c–f) show severe central vein (CV) and portal vein (PV) congestion, moderate congestion of sinusoidal capillaries (d), and marked hydropic degeneration (black arrows in (f)). Light microscopic sections of the liver in the CAT-only group (g, h) show typical organized liver structures, an uncongested CV with mild sinusoidal congestion (yellow arrows). Light microscopic liver sections in PAC + DOX + high CAT dose group (i, j) display mild central vein and portal vein congestion, moderate cellular swelling, and minimal neutrophil infiltration in perilobular regions as indicated by black arrows. Light microscopic sections of the liver in the PAC + DOX + high CAT dose group (k, l) show moderate CV and PV congestion, with mild congestion in the sinusoidal capillary (yellow arrows), moderate hepatocyte swelling, and mild infiltration of neutrophil in centrilobular regions and perilobular regions as indicated by black arrows (H&E stain).

**Figure 6:**
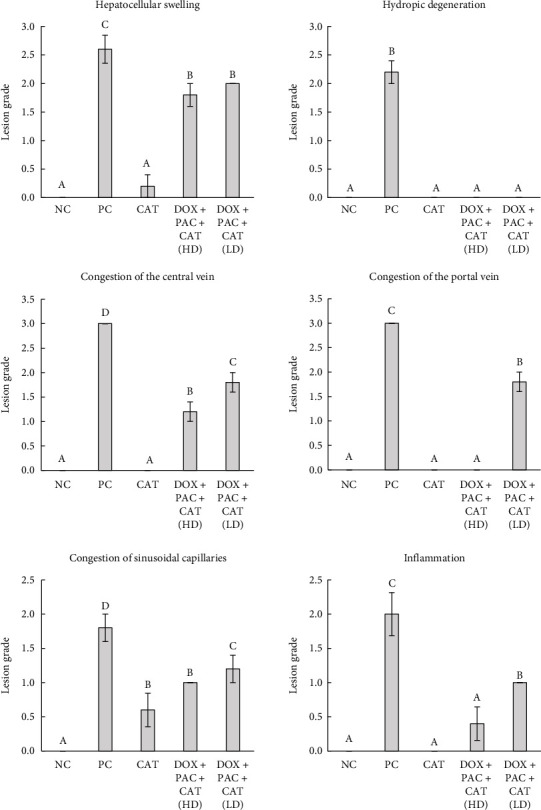
Grading of lesions across the experimental groups. Scores are shown as columns, while the error bars indicate standard errors of the means. Groups marked with different letters differ significantly at *p* < 0.05. Different letters indicate statistical differences between the groups, as determined by the Kruskal–Wallis one-way ANOVA test.

The NC group's microscopic liver section showed normal histologic organization and features, including well-organized hepatic lobules with the central vein surrounded by plates of intact hepatocytes. At each hepatic periphery, there are typical structures of the portal area, such as the hepatic artery branches, portal vein, and hepatic bile duct, without any indications of inflammation or circulatory disturbance (Figures 5(a) and 5(b)). However, compared to the rats in the NC group, the liver sections of the PC rats showed severe hepatic changes (acute hepatic congestion) and a high lesion score. These changes included enlarged, pale vacuole cells with eccentrically located nuclei, called hydropic degeneration, marked congestion of the central and portal veins, moderate sinusoidal capillary congestion, considerable hepatocyte swelling, and liver cell enlargement with a centrally located nucleus, and narrowed sinusoidal capillaries. Additionally, there was moderate neutrophil infiltration, especially in the periportal and central vein regions (Figures 5(c), 5(d), 5(e), and 5(f)). The histopathological grading of hepatic tissue is shown in Figure 6.

Administration of CAT did not alter the liver parenchyma compared to the PC group and showed a well-organized hepatic lobule structure with a mild degree of congestion only in the sinusoids (Figures 5(g) and 5(h)). The administration of the high CAT dose attenuated the effect of PAC + DOX, revealing focal mild congestion of the central vein. At the same time, the portal area remained normal, with mild congestion of sinusoidal capillaries, moderate swelling of hepatocytes, and mild inflammatory reaction in the periportal region (Figures 5(i) and 5(j)) compared to the PC group. Additionally, administering the low dose of CAT with PAC + DOX improved the liver lesions and reduced the pathologic scores. Moderate congestion of the central and portal veins was observed, with mild sinusoidal congestion, moderate cloudy swelling of liver cells, and mild inflammatory reaction in the centrilobular and periportal regions (Figures 5(k) and 5(l)).

## 4. Discussion

The present study provides substantial evidence that the administration of DOX and PAC induces hepatotoxicity, as demonstrated by increased liver weights, elevated liver function biomarkers, oxidative stress, and histopathological alterations. Coadministration of CAT, particularly at a dose of 40 mg/kg, effectively mitigated these adverse effects, reinforcing its hepatoprotective potential. The findings of this study align with previous literature, which suggests that CAT exerts antioxidant and anti-inflammatory properties capable of counteracting chemotherapy-induced organ toxicity.

Hepatotoxicity induced by DOX and PAC is primarily attributed to their ability to generate ROS, leading to oxidative stress, mitochondrial dysfunction, and inflammation. DOX, an anthracycline, intercalates into DNA and disrupts topoisomerase II, which, in addition to exerting anticancer effects, also causes mitochondrial damage and lipid peroxidation. Similarly, PAC, a taxane, disrupts microtubule function and has been shown to enhance ROS production, further exacerbating cellular damage [16, 31]. These mechanisms increase the release of liver enzymes into the bloodstream, as observed in the PC group.

The significant reduction in ALT, AST, and ALP levels following 40 mg/kg CAT administration suggests that CAT counteracts these oxidative effects by enhancing endogenous antioxidant defense mechanisms. This aligns with findings from previous studies, which demonstrate that CAT can scavenge free radicals, inhibit lipid peroxidation, and upregulate the activity of antioxidant enzymes, including SOD and catalase [28, 32]. The observed increase in GSH and catalase levels in the high-dose CAT group further supports the role of CAT in strengthening the hepatic antioxidant system.

Inflammation plays a crucial role in chemotherapy-induced hepatotoxicity [33]. NF-κB, a key transcription factor in inflammatory responses [34], was significantly elevated in the PC group, suggesting that DOX and PAC induced hepatic inflammation. CAT administration, particularly at a dose of 40 mg/kg, significantly reduced NF-κB expression, indicating an anti-inflammatory effect. This effect is consistent with previous research showing that CAT inhibits proinflammatory cytokines, including tumor necrosis factor-alpha (TNF-α) and interleukin-6 (IL-6) [35, 36], thereby preventing inflammatory liver damage.

Hematological findings further revealed the adverse effects of DOX and PAC on leukocyte and thrombocyte counts. Myelosuppression is a well-known side effect of these chemotherapeutic agents, as they target rapidly dividing cells, including hematopoietic progenitors. While CAT did not fully prevent leukocytopenia, its protective effects on thrombocyte counts suggest a partial cytoprotective role. Future studies should explore how CAT influences hematopoietic recovery in chemotherapy-treated patients.

Histopathological analysis reinforced the biochemical findings, with the PC group displaying severe hepatic damage, including congestion, hepatocyte degeneration, and inflammatory infiltration. The protective effects of CAT were evident in the 40 mg/kg CAT group, as indicated by histological examination, which showed reduced congestion, mild hepatocyte swelling, and minimal inflammatory cell infiltration. These observations suggest that CAT effectively attenuates histopathological damage, further validating its hepatoprotective role.

The findings of this study have important clinical implications. Given that hepatotoxicity is a major dose-limiting toxicity in cancer treatment, incorporating hepatoprotective agents such as CAT could enhance the therapeutic index of chemotherapeutic regimens. Future studies should focus on determining the optimal dosage and administration schedule of CAT in combination with chemotherapy to maximize its protective effects while ensuring its safety and efficacy.

Moreover, the precise molecular mechanisms by which CAT exerts its protective effects warrant further investigation. Emerging research suggests that CAT may modulate key signaling pathways involved in oxidative stress, apoptosis, and inflammation, including the nuclear factor erythroid 2-related factor 2 (Nrf2) pathway and mitogen-activated protein kinase (MAPK) signaling [37]. Understanding these mechanisms could facilitate the development of targeted hepatoprotective strategies in oncology.

## 5. Conclusion

This study provides compelling evidence that CAT, particularly at a dose of 40 mg/kg, exhibits significant hepatoprotective effects against DOX- and PAC-induced hepatotoxicity. By reducing oxidative stress, inflammation, and hepatocellular damage, CAT demonstrates potential as a supportive therapeutic agent in chemotherapy. Future clinical studies are necessary to validate these findings and assess the feasibility of CAT supplementation in cancer patients undergoing chemotherapy.

## Figures and Tables

**Table 1:** Assessment of liver lesions in the studied groups.

Histopathologic abnormalities	Grade	Interpretation
Congestion and hemorrhage	0	Absence of change
	1	Change in 1%–25%
	2	Change in 26%–50%
	3	Change in 51%–75%
	4	Change in > 75%

Hepatocyte swelling and hydropic degeneration	0	Absence of change
	1	Change in 1%–25%
	2	Change in 26%–50%
	3	Change in 51%–75%
	4	Change in > 75%

Inflammation of the parenchyma	0	Absence of change
	1	Change in 1%–25%
	2	Change in 26%–50%
	3	Change in 51%–75%
	4	Change in > 75%

*Note:* Adapted from Hassan et al. [30].

**Table 2:** Total and differential leukocyte counts.

Group	Leukocytes (no. × 10^3^/μL)	Lymphocytes (%)	Neutrophils (%)	Monocytes (%)	Eosinophils (%)	Basophils (%)
NC	7.38^b^ ± 0.94	74.48^b^ ± 2.42	16.02^a^ ± 2.47	9.17^a^ ± 2.93	0.33^a^ ± 0.07	0.020^a^ ± 0.004
PC	2.51^a^ ± 1.00	51.05^a^ ± 6.06	41.25^b^ ± 6.97	7.35^a^ ± 2.91	0.28^a^ ± 0.09	0.010^a^ ± 0.000
CAT	10.16^c^ ± 1.46	71.35^b^ ± 6.83	19.65^a^ ± 3.20	8.68^a^ ± 3.85	0.33^a^ ± 0.09	0.035^b^ ± 0.013
DOX + PAC + CAT (HD)	2.06^a^ ± 0.36	47.43^a^ ± 5.00	42.42^b^ ± 7.62	9.15^a^ ± 2.31	0.85^a^ ± 0.53	0.012^a^ ± 0.002
DOX + PAC + CAT (LD)	1.68^a^ ± 0.35	58.40^ab^ ± 7.14	31.08^ab^ ± 7.04	9.78^a^ ± 2.83	0.68^a^ ± 0.51	0.008^a^ ± 0.002

*Note:* Values represent the means of six rats per group ± SEM. Different letters (a, b, c) denote significant differences within the same column at *p* < 0.05.

**Table 3:** Hematological parameters.

Group	Erythrocytes (no. × 10^6^/μL)	Hemoglobin (g/dL)	Hematocrit (%)	MCV (fL)	MCH (pg)	MCHC (%)	Thrombocytes (no. × 10^6^/μL)
NC	7.15^a^ ± 0.24	15.03^a^ ± 0.38	45.38^a^ ± 1.70	63.53^a^ ± 1.40	21.05^a^ ± 0.23	33.23^a^ ± 0.72	631.83^b^ ± 47.81
PC	6.66^a^ ± 0.15	14.67^a^ ± 0.35	45.43^a^ ± 1.73	68.27^ab^ ± 2.16	22.03^a^ ± 0.15	32.43^a^ ± 1.12	417.67^a^ ± 43.71
CAT	6.78^a^ ± 0.21	14.93^a^ ± 0.38	46.03^a^ ± 2.40	67.88^ab^ ± 2.59	22.08^a^ ± 0.41	32.63^a^ ± 1.00	596.75^b^ ± 26.96
DOX + PAC + CAT (HD)	7.19^a^ ± 0.22	15.70^a^ ± 0.28	48.23^a^ ± 1.60	67.15^ab^ ± 1.08	21.90^a^ ± 0.29	32.63^a^ ± 0.67	480.33^a^ ± 18.79
DOX + PAC + CAT (LD)	6.81^a^ ± 0.23	14.76^a^ ± 0.49	51.02^a^ ± 2.67	71.26^b^ ± 3.05	21.74^a^ ± 0.69	29.40^a^ ± 2.11	453.33^a^ ± 38.23

*Note:* Values represent the means of six rats per group ± SEM. Different letters (a, b) denote significant differences within the same column at *p* < 0.05.

## Data Availability

The data that support the findings of this study are available upon request from the corresponding author.
